# The deubiquitinating enzyme UCHL1 negatively regulates the immunosuppressive capacity and survival of multipotent mesenchymal stromal cells

**DOI:** 10.1038/s41419-018-0532-y

**Published:** 2018-04-23

**Authors:** Yuting Gu, Xinyuan Ding, Jiefang Huang, Mingxing Xue, Jie Zhang, Qiwei Wang, Hongshuang Yu, Yanan Wang, Fang Zhao, Hui Wang, Min Jin, Yeming Wu, Yanyun Zhang

**Affiliations:** 1 Shanghai Institute of Immunology, Shanghai Jiao Tong University School of Medicine, Shanghai, China; 2 Shanghai Institutes for Biological Sciences, Chinese Academy of Sciences, Shanghai, China; 3 Department of Pediatric Surgery, Xinhua Hospital Affiliated to Shanghai Jiao Tong University School of Medicine, Shanghai, China; 4 Department of Pharmacy, The Affiliated Suzhou Hospital of Nanjing Medical University, Suzhou, China; 5 Pediatric Institute of Soochow University, Institutes for Translational Medicine, Soochow University, Suzhou, China

## Abstract

It is known that proinflammatory cytokines empower multipotent mesenchymal stromal cells (MSCs) the immunosuppressive capacity to treat various inflammatory diseases. Nevertheless, how the proinflammatory cytokines modulate the immunosuppressive capacity of MSCs is poorly understood. In the present study, we identified that the deubiquitinating enzyme ubiquitin C-terminal hydrolase 1 (UCHL1) was upregulated in MSCs upon stimulation of proinflammatory cytokines IFN-γ plus TNF-α. Interestingly, through intervening UCHL1 by shRNA knockdown or its inhibitor LDN57444 or overexpression, we found that UCHL1 played a critical role in suppressing cytokines-induced inducible nitric oxide synthase expression in murine MSCs and indoleamine 2,3-dioxygenase expression in human MSCs, thereby restrained their immunosuppressive capacity. This effect of UCHL1 was attributed to the negative role in regulating NF-κB and STAT1 signaling, as exhibited by promoting NF-κB and STAT1 activation upon inhibition of UCHL1. Besides, inhibition of UCHL1 suppressed cytokines-induced MSC apoptosis via upregulation of Bcl-2. As a consequence, UCHL1-inhibited MSCs effectively alleviated concanavalin A-induced inflammatory liver injury. Therefore, our study demonstrates a novel role of UCHL1 in regulating the immunosuppressive capacity and survival of MSCs, which further affects their immunotherapy for inflammatory diseases.

## Introduction

Multipotent mesenchymal stromal cells (MSCs) are one kind of adult progenitor cells, which exist in various tissues and can be isolated from bone marrow, fat, muscle, teeth, and skin. Initially, MSCs are always investigated for their self-renewal and multilineage differentiation potential for regenerative medicine^1,2^. Emerging evidences have shown that MSCs hold the capacity of immunosuppression and therapeutic potential for various inflammatory diseases^1,3^. MSCs can secrete a serious of immunosuppressive molecules and chemokines, such as nitric oxide (NO), indoleamine 2, 3-dioxygenase (IDO), prostaglandin E2 (PGE2), transforming growth factor β, tumor necrosis factor-inducible gene 6, interleukin-6 and chemokine (C-X-C motif) ligand 9 (CXCL9), and express several surface ligands, such as FAS ligand (FasL), CD112, and CD155, to further inhibit the proliferation and functions of immune cells, including dendritic cells, T and B lymphocytes to alleviate the severity of inflammatory diseases^4–7^.

Proinflammatory cytokines such as IFN-γ, TNF-α, and IL-1β are critical for inducing the immunosuppressive capacity of MSCs^8^. These proinflammatory cytokines not only induce the immunosuppressive capacity of MSCs, but also have their adverse effects. Previous studies showed that microRNA-155 and suppressor of cytokine signaling 1 were induced by proinflammatory cytokines, both of which inhibited the immunosuppressive capacity of MSCs on T cell proliferation by reducing inducible nitric oxide synthase (iNOS) expression^9,10^. Our previous studies also showed that inflammatory microenvironment-induced autophagy in MSCs, which dampened their immunosuppressive capacity and therapeutic effects on inflammatory diseases^11,12^. In addition, IFN-γ plus TNF-α could induce the apoptosis of MSCs, which inhibited their therapeutic effects on bone repair^13^. Thus, a better understanding of the mechanisms by which the inflammatory microenvironment regulates the immunosuppressive capacity and survival of MSCs is needed to guide future clinical use of MSCs.

Ubiquitination and deubiquitination, the reversible post-translational modification of protein relied on the ubiquitin ligases and deubiquitinating (DUB) enzymes, are involved in nearly all areas of cell biology, which lies in their capacity to determine protein stability, functional activation, and subcellular localization^14–16^. In MSCs, most studies on the ubiquitination and deubiquitination have focused on their roles in differentiation^17–20^, but whether this process can regulate the immunosuppressive capacity of MSCs is still elusive. Ubiquitin C-terminal hydrolase 1 (UCHL1), a DUB enzyme, is one of the important members of the UCH family that catalyze the hydrolysis of COOH-terminal ubiquityl esters and amides^21,22^. UCHL1 can induce apoptosis of tumor cells and is involved in various cancers^23–26^. Recent studies also have found that UCHL1 plays critical roles in immune responses and MSC-associated giant cell tumor^27,28^. In view of these findings, we set out to examine a possible role of UCHL1 in regulating proinflammatory cytokines-induced immunosuppressive capacity or survival of MSCs.

In the present study, we identified that UCHL1 was upregulated in MSCs upon proinflammatory cytokines IFN-γ plus TNF-α stimulation. Interestingly, inhibition of UCHL1 significantly improved the immunosuppressive capacity both of murine and human MSCs, characterized by increased iNOS and IDO expression, respectively. This effect of UCHL1 was exerted by negatively regulating cytokines-induced NF-κB and STAT1 signaling activation. In addition, we found that inhibition of UCHL1 upregulated the expression of B-cell lymphoma-2 (Bcl-2), which in turn suppressed proinflammatory cytokines-induced MSC apoptosis. As a consequence, UCHL1-inhibited MSCs effectively alleviated concanavalin A (ConA)-induced inflammatory liver injury. Therefore, our study demonstrates a novel function of UCHL1 in regulating the immunosuppressive capacity and survival of MSCs, which further controls their immunotherapy for inflammatory diseases.

## Results

### Proinflammatory cytokines upregulates UCHL1 expression in murine MSCs

**Fig. 1:**
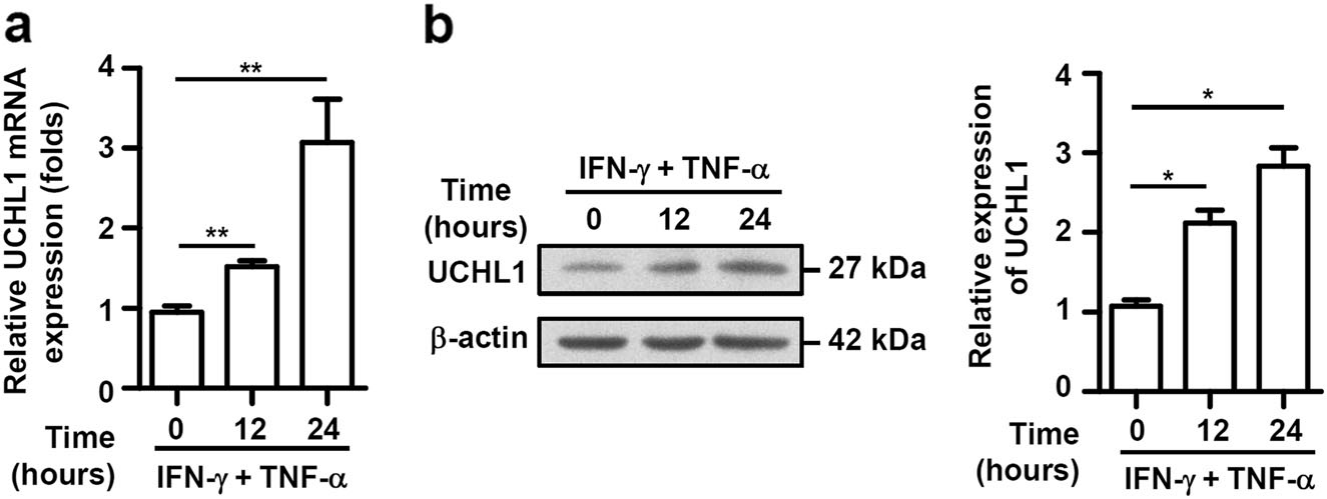
IFN-γ plus TNF-α upregulated UCHL1 expression in murine MSCs. **a**, **b** Murine MSCs were treated with IFN-γ plus TNF-α (10 ng/ml each) for 12 and 24 h, mRNA and protein were collected. UCHL1 expression was determined at the mRNA and protein levels by quantitative real-time PCR and immunoblotting analysis. Values are shown as mean ± S.E.M. and statistical significance indicated as *P < 0.05 and **P < 0.01.

Proinflammatory cytokines, such as IFN-γ, TNF-α, and IL-1β, are critical for inducing the immunosuppressive capacity of MSCs^8,11^. To determine whether UCHL1 was involved in regulating the immunosuppressive capacity of MSCs, we examined the expression of UCHL1 in murine MSCs before and after IFN-γ plus TNF-α stimulation. Similar to the immunosuppressive factors, such as iNOS, PGE2, prostaglandin-endoperoxide synthase 2 (PTGS2), CXCL10, CXCL9, etc. (data not shown), both the mRNA and protein levels of UCHL1 dramatically increased in murine MSCs after stimulation of IFN-γ plus TNF-α (Fig. 1a, b), suggesting a potential role of UCHL1 in regulating the immunosuppressive capacity of MSCs.

### UCHL1 negatively regulates the immunosuppressive capacity of murine MSCs

**Fig. 2:**
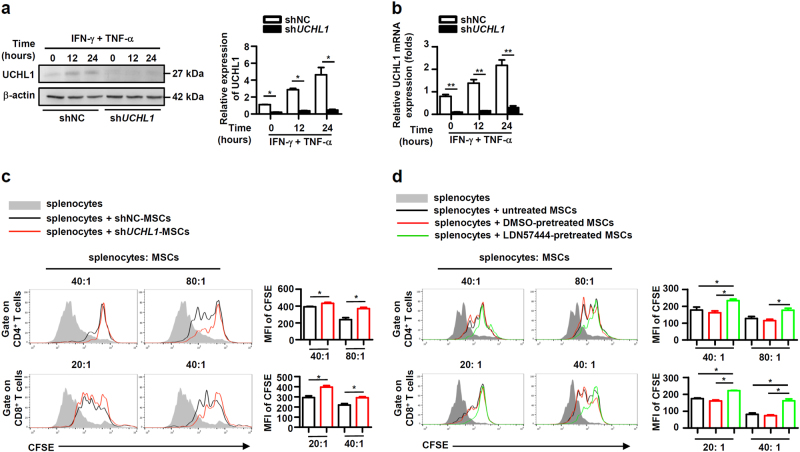
Inhibition of UCHL1 enhanced the ability of murine MSCs to suppress T cell proliferation. **a**, **b** Murine MSCs were infected with control lentivirus (shNC-MSCs) or lentivirus expressing shRNA targeting UCHL1 (sh*UCHL1*-MSCs), and treated with IFN-γ plus TNF-α (10 ng/ml each) for 12 and 24 h. UCHL1 expression was measured by immunoblotting analysis and quantitative real-time PCR. **c** Irradiated shNC-MSCs or sh*UCHL1*-MSCs were co-cultured with CFSE-labeled splenocytes for 3 days in the presence of anti-CD3/CD28 antibodies at the indicated ratios. CD8^+^ and CD4^+^ T cells were collected for proliferation analysis by flow cytometry at the end of co-culture and median fluorescence intensity (MFI) of CFSE of T cells were shown. **d** Murine MSCs were pretreated with DMSO or LDN57444 (10 μM) for 24 h, and irradiated untreated, DMSO-pretreated or LDN57444-pretreated murine MSCs were co-cultured with CFSE-labeled splenocytes for 3 days in the presence of anti-CD3/CD28 antibodies at the indicated ratios. CD8^+^ and CD4^+^ T cells were collected for proliferation analysis by flow cytometry at the end of co-culture and MFI of CFSE of T cells were shown. Values are shown as mean ± S.E.M. and statistical significance indicated as *P < 0.05 and **P *<* 0.01.

**Fig. 3:**
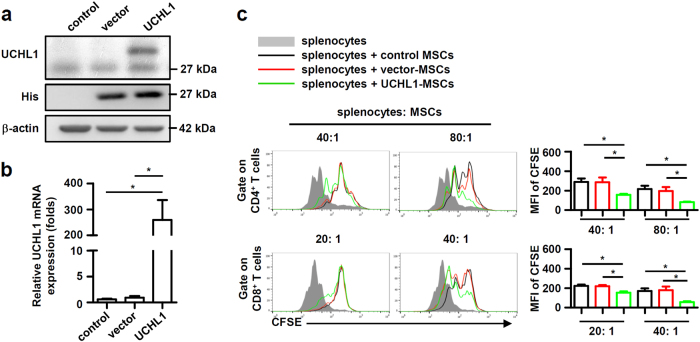
UCHL1 overexpression inhibited the ability of murine MSCs to suppress T cell proliferation. **a**, **b** Murine MSCs were transfected with pcDNA3.1 (vector-MSCs) or pcDNA3.1 containing UCHL1 (UCHL1-MSCs), and UCHL1 expression was measured by immunoblotting analysis and quantitative real-time PCR. **c** Irradiated control MSCs, vector-MSCs and UCHL1-MSCs were co-cultured with CFSE-labeled splenocytes for 3 days in the presence of anti-CD3/CD28 antibodies at the indicated ratios. CD8^+^ and CD4^+^ T cells were collected for proliferation analysis by flow cytometry at the end of co-culture and MFI of CFSE of T cells were shown. Values are shown as mean ± S.E.M. and statistical significance indicated as *P < 0.05.

To study the role of UCHL1 in regulating the immunosuppressive capacity of MSCs, UCHL1 was inhibited or overexpressed in murine MSCs to examine their effects on T cell proliferation. As shown in Fig. 2a-c, knockdown of UCHL1 in murine MSCs by using a lentivirus expressing shRNA specific to UCHL1 (hereafter called sh*UCHL1*-MSCs) suppressed the proliferation of activated T cells more efficiently than the MSCs infected with a lentivirus expressing scrambled shRNA (hereafter called shNC-MSCs). To further confirm the function of UCHL1, LDN57444, a reversible, competitive, active-site directed inhibitor of UCHL1^29^, was used to examine the regulation of UCHL1 on MSC immunosuppression. Compared with untreated and DMSO-pretreated MSCs, LDN57444-pretreated MSCs held enhanced immunosuppressive effects on T cell proliferation (Fig. 2d). In contrast, UCHL1 overexpression in MSCs dramatically suppressed their immunosuppressive effects on T cell proliferation (Fig. 3a-c). Collectively, these results identify a critical role of UCHL1 in negatively regulating the immunosuppressive capacity of MSCs.

### UCHL1 inhibition enhances iNOS expression in murine MSCs

**Fig. 4:**
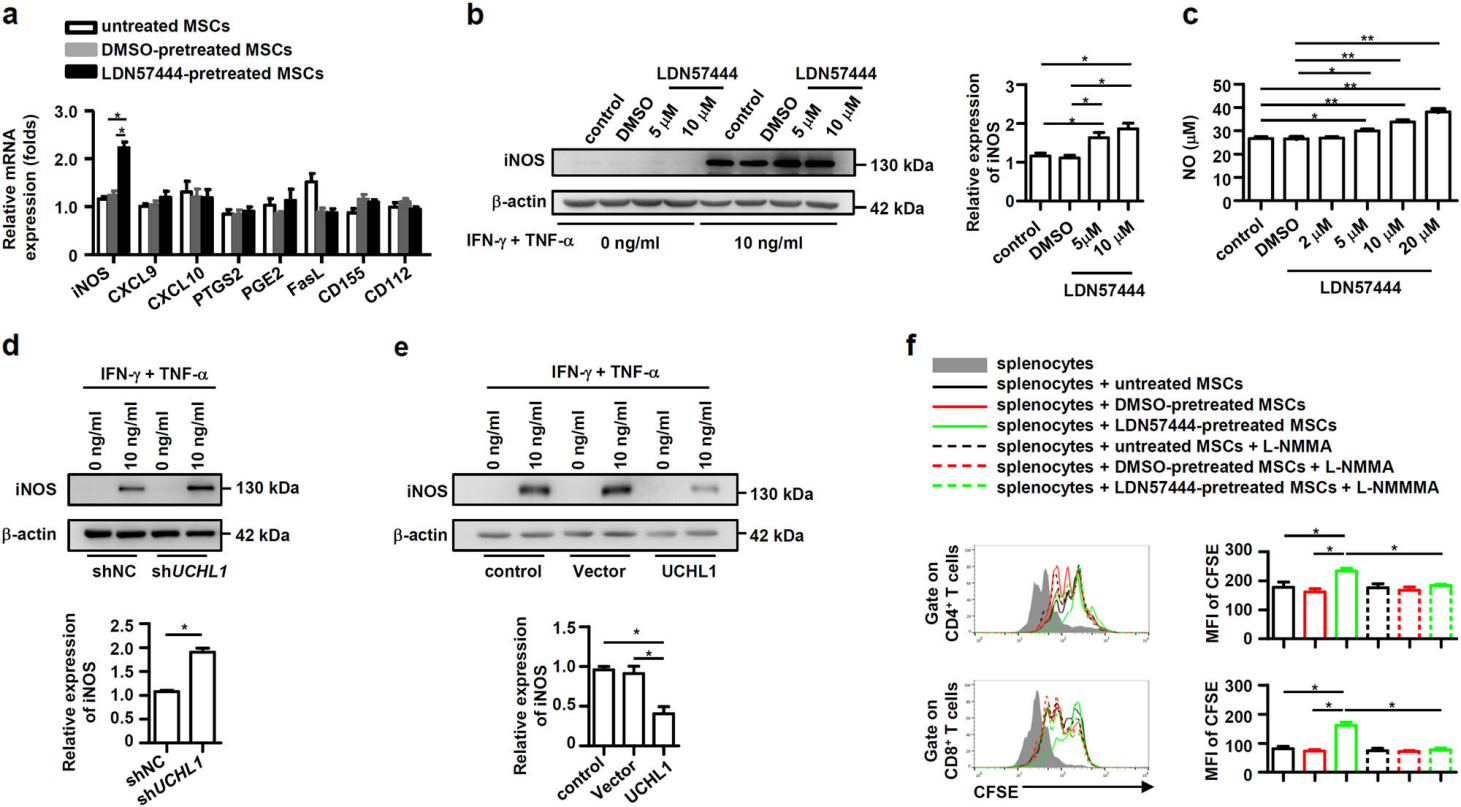
UCHL1 inhibition enhanced iNOS expression in murine MSCs. **a** Murine MSCs were pretreated with DMSO or LDN57444 (10 μM) for 24 h and then treated with IFN-γ plus TNF-α (10 ng/ml each) for 12 h. The mRNA expression levels of iNOS, CXCL9, CXCL10, PTGS2, FasL, CD155 and CD112 were measured by quantitative real-time PCR. **b**, **c** MSCs were pretreated with DMSO or LDN57444 for 24 h and then treated with or without IFN-γ plus TNF-α (10 ng/ml each) for 24 h. Expression of iNOS protein and the supernatant NO concentration were examined by immunoblotting analysis and Griess assay. **d** shNC-MSCs or sh*UCHL1*-MSCs were treated with or without IFN-γ plus TNF-α (10 ng/ml each) for 24 h. Expression of iNOS protein were examined by immunoblotting analysis. **e** Control MSCs, vector-MSCs and UCHL1-MSCs were treated with or without IFN-γ plus TNF-α (10 ng/ml each) for 24 h. Expression of iNOS protein was examined by immunoblotting analysis. **f** Murine MSCs were pretreated with DMSO or LDN57444 (10 μM) for 24 h, irradiated untreated, DMSO-pretreated or LDN57444-pretreated murine MSCs were co-cultured with CFSE-labeled splenocytes for 3 days in the presence of anti-CD3/CD28 antibodies at the ratio of 40: 1. L-NMMA was added to block iNOS activity. CD8^+^ and CD4^+^ T cells were collected for proliferation analysis by flow cytometry at the end of co-culture and MFI of CFSE of T cells were shown. Values are shown as mean ± S.E.M. and statistical significance indicated as *P < 0.05 and **P *<* 0.01.

It has been well characterized that the immunosuppressive molecules, chemokines, and surface ligands, such as iNOS, CXCL9, CXCL10, PTGS2, PGE2, FasL, CD155, CD112, etc., are essential for the immunosuppressive effects of MSCs on T cell proliferation^7,8^. To determine the underlying mechanisms of UCHL1-mediated regulation of MSC immunosuppression, murine MSCs were pretreated with LDN57444 and then stimulated with IFN-γ plus TNF-α to detect the expression of these immunosuppressive factors. The results revealed that LDN57444 pretreatment did not affect the gene expression of CXCL9, CXCL10, PTGS2, PGE2, FasL, CD155, and CD112 whereas notably enhanced iNOS expression at both mRNA and protein levels (Fig. 4a, b). Accordingly, NO, a downstream product of iNOS in murine MSCs and an effector of immunosuppression, significantly increased in the supernatant fraction of LDN57444-pretreated MSCs in a dose dependent manner (Fig. 4c). Besides, compared with shNC-MSCs, sh*UCHL1*-MSCs also exhibited upregulated iNOS expression after IFN-γ plus TNF-α stimulation (Fig. 4d), whereas UCHL1 overexpression inhibited iNOS expression (Fig. 4e). Furthermore, a functional study showed that N^G^-monomethyl-L-arginine (L-NMMA), a specific inhibitor of iNOS activity, reversed the enhanced suppressive effects of LDN57444-pretreated MSCs on T cell proliferation (Fig. 4f). Therefore, these data collectively suggest that inhibition of UCHL1 enhances the immunosuppressive capacity of MSCs mainly through upregulating iNOS expression.

### UCHL1 inhibition promotes the activation of NF-κB and STAT1 signaling

**Fig. 5:**
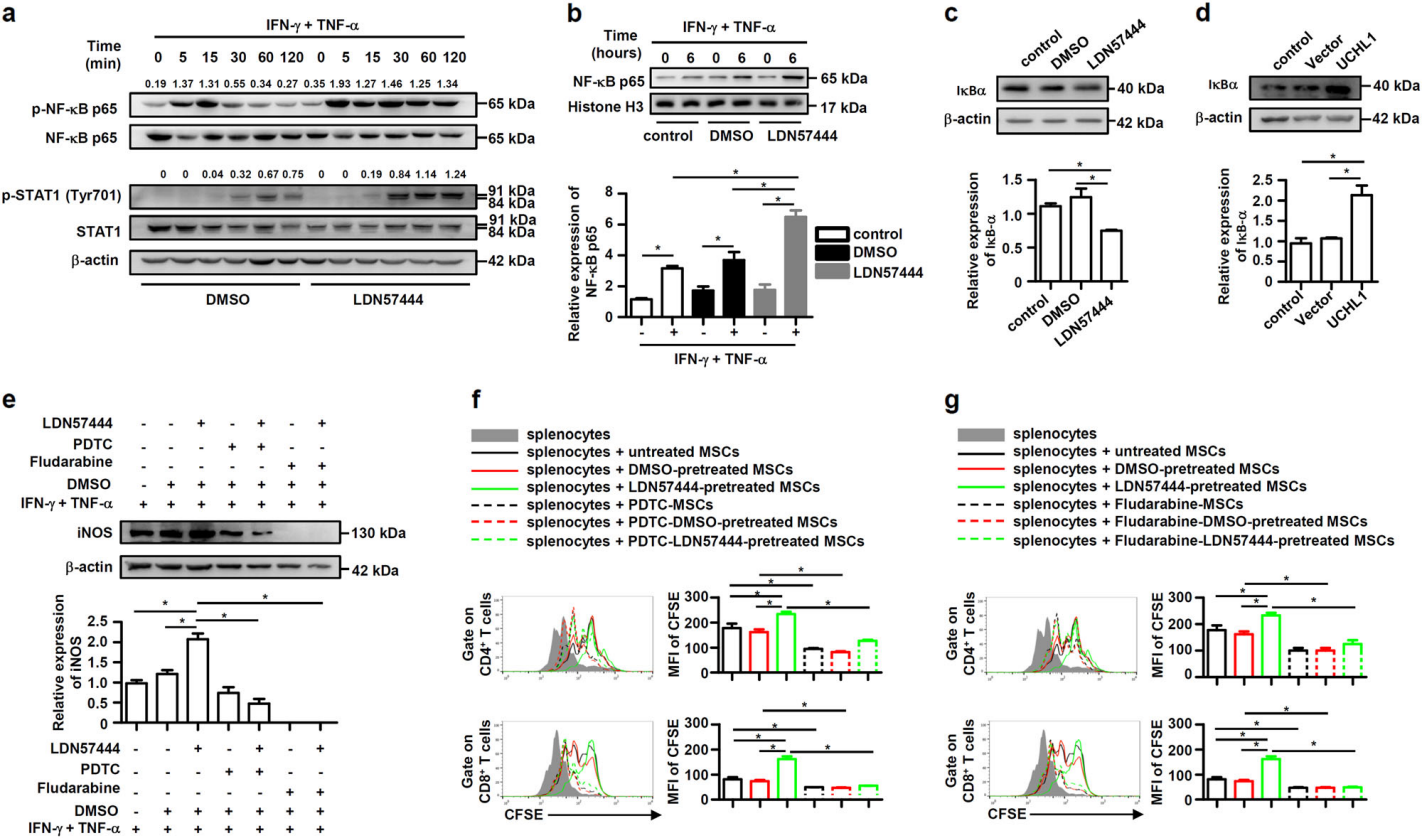
UCHL1 inhibition promoted the activation of NF-κB and STAT1 signaling. **a** After pretreatment with DMSO or LDN57444 (10 μM), murine MSCs were treated with IFN-γ plus TNF-α (10 ng/ml each) for the indicated time. Cells were harvested and NF-κB p65, STAT1, phosphorylation of NF-κB p65 and STAT1 at Tyr701 were analyzed by immunoblotting analysis. The densitometry of p-NF-κB p65 and p-STAT1 (Tyr701) was quantified using ImageJ software, and NF-κB p65 and STAT1 were used as controls, respectively. **b** After pretreatment with DMSO or LDN57444 (10 μM) for 24 h, murine MSCs were treated with or without IFN-γ plus TNF-α (10 ng/ml each) for 6 h. Nucleus protein of cells was harvested and NF-κB p65 was analyzed by immunoblotting analysis. **c** After pretreatment with DMSO or LDN57444 (10 μM) for 24 h, cells were harvested and IκBα expression was analyzed by immunoblotting analysis. **d** Control MSCs, vector-MSCs and UCHL1-MSCs were harvested, and IκBα expression was analyzed by immunoblotting analysis. **e** After pretreatment with DMSO or LDN57444 (10 μM) for 24 h, murine MSCs were pretreated with NF-κB inhibitor PDTC (1 μM) or STAT1 inhibitor Fludarabine (2 μM) for 6 h prior to IFN-γ plus TNF-α (10 ng/ml each) treatment for 24 h. Cells were harvested and iNOS expression was analyzed by immunoblotting analysis. **f**, **g** After pretreatment with DMSO or LDN57444 (10 μM) for 24 h, irradiated untreated, DMSO-pretreated or LDN57444-pretreated murine MSCs were pretreated with PDTC (1 μM) or Fludarabine (2 μM) for 6 h prior to co-culture with CFSE-labeled splenocytes for 3 days in the presence of anti-CD3/CD28 antibodies at the ratio of 40: 1. CD8^+^ and CD4^+^ T cells were collected for proliferation analysis by flow cytometry at the end of co-culture and MFI of CFSE of T cells were shown. Values are shown as mean ± S.E.M. and statistical significance indicated as *P < 0.05.

NF-κB and STAT1 pathway are critical signalings for IFN-γ plus TNF-α-induced iNOS expression^30^. As UCHL1 was reported to regulate the canonical NF-κB signaling^28,31^, we firstly performed immunoblotting analysis to determine cytokines-induced NF-κB activation upon UCHL1 intervention in MSCs. As shown in Fig. 5a, b, LDN57444 pretreatment dramatically promoted the phosphorylation of NF-κB p65 and the nuclear NF-κB p65 translocation in MSCs after IFN-γ plus TNF-α stimulation. Additionally, LDN57444 pretreatment reduced the protein levels of IκBα, the negative NF-κB modulator (Fig. 5c), while overexpression of UCHL1 maintained IκBα levels (Fig. 5d), suggesting that UCHL1 promoted the stability of IκBα, which may further inhibit NF-κB activation in MSCs. Moreover, IFN-γ plus TNF-α-induced phosphorylation of STAT1 at Tyr701 was also enhanced in MSCs after LDN57444 treatment (Fig. 5a). Functionally, pretreatment with the NF-κB inhibitor pyrrolidine dithiocarbamate (PDTC) or the STAT1 inhibitor Fludarabine reversed the increased iNOS expression (Fig. 5e), and abolished the enhanced suppressive effects on T cell proliferation (Fig. 5f, g) of LDN57444-pretreated MSCs. These data suggest that inhibition of UCHL1 promotes NF-κB and STAT1 activation, which in turn enhances iNOS expression and the immunosuppressive capacity of murine MSCs.

### UCHL1 inhibition promotes the immunosuppressive capacity and IDO expression of human MSCs

**Fig. 6:**
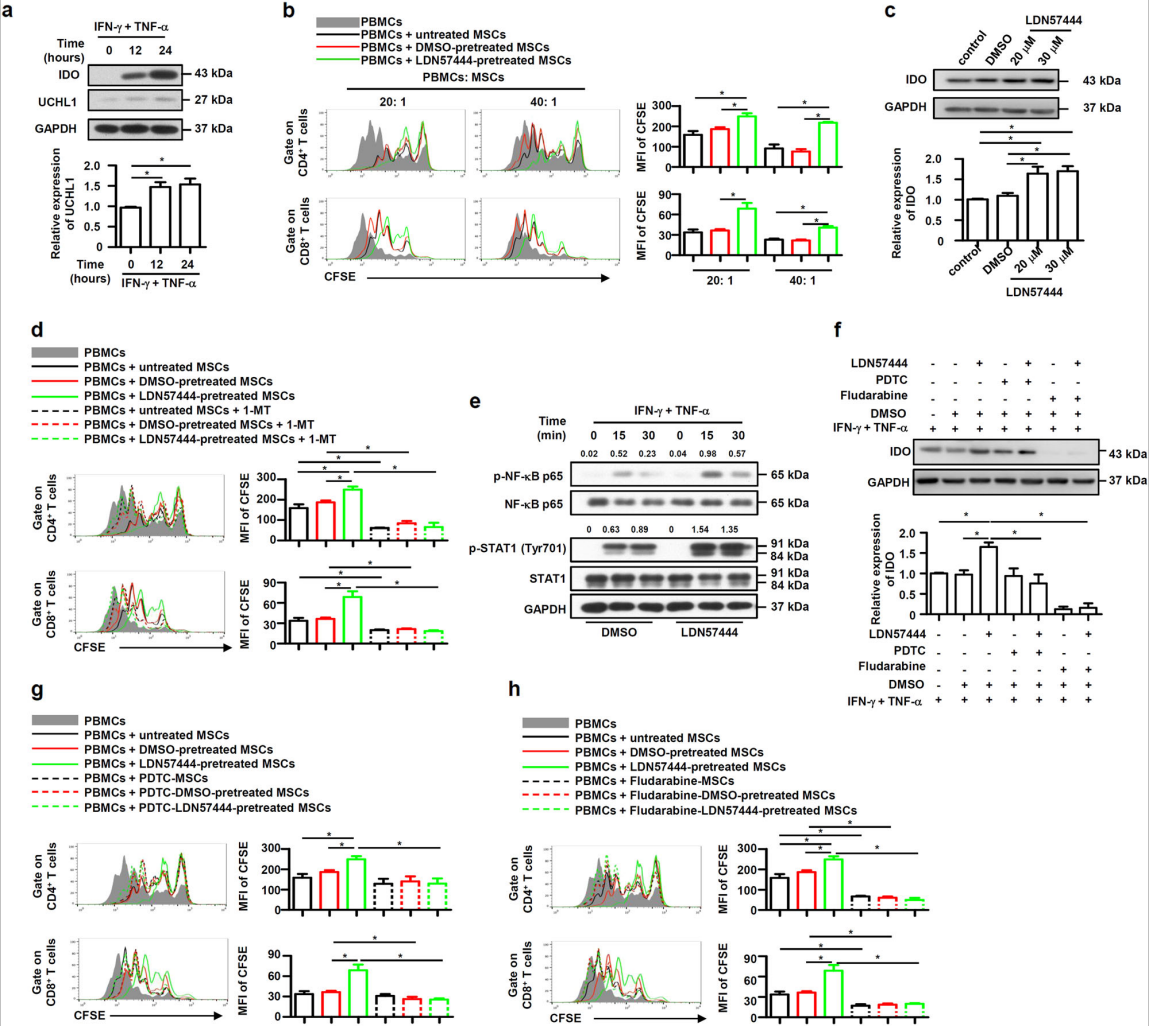
UCHL1 inhibition promoted the immunosuppressive capacity and IDO expression of human MSCs. **a** Human MSCs were treated with human IFN-γ plus TNF-α (5 ng/ml each) for 12 and 24 h, and UCHL1 expression was examined by immunoblotting analysis. **b** Human MSCs were pretreated with DMSO or LDN57444 (20 μM) for 24 h, and irradiated untreated, DMSO-pretreated or LDN57444-pretreated human MSCs were co-cultured with CFSE-labeled human PBMCs for 3 days in the presence of anti-CD3/CD28 antibodies at the indicated ratios. CD8^+^ and CD4^+^ T cells were collected for proliferation analysis by flow cytometry at the end of co-culture and MFI of CFSE of T cells were shown. **c** After pretreatment with DMSO or LDN57444 (20 μM or 30 μM) for 24 h, human MSCs were treated with human IFN-γ plus TNF-α (5 ng/ml each) for 24 h, and IDO expression was examined by immunoblotting analysis. **d** After pretreatment with DMSO or LDN57444 (20 μM) for 24 h, irradiated human MSCs were co-cultured with CFSE-labeled human PBMCs for 3 days in the presence of anti-CD3/CD28 antibodies at the ratio of 40: 1, and 1-MT was added to block IDO. CD8^+^ and CD4^+^ T cells were collected for proliferation analysis by flow cytometry at the end of co-culture and MFI of CFSE of T cells were shown. **e** After pretreatment with DMSO or LDN57444 (20 μM) for 24 h, human MSCs were treated with human IFN-γ plus TNF-α (5 ng/ml each), phosphorylation of NF-κB p65 and STAT1 at Tyr701 were examined by immunoblotting analysis at the indicated time points. The densitometry of p-NF-κB p65 and p-STAT1 (Tyr701) was quantified using ImageJ software, and NF-κB p65 and STAT1 were used as controls, respectively. **f** After pretreatment with DMSO or LDN57444 (20 μM), human MSCs were pretreated with PDTC (1 μM) or Fludarabine (2 μM) for 6 h prior to IFN-γ plus TNF-α (5 ng/ml each) treatment for 24 h, and IDO expression was examined by immunoblotting assay. **g**, **h** After pretreatment with DMSO or LDN57444 (20 μM) for 24 h, irradiated human MSCs were pretreated with PDTC (1 μM) or Fludarabine (2 μM) for 6 h prior to co-culture with human PBMCs for 3 days in the presence of anti-CD3/CD28 antibodies at the ratio of 40: 1. CD8^+^ and CD4^+^ T cells were collected for proliferation analysis by flow cytometry at the end of co-culture and MFI of CFSE of T cells were shown. Values are shown as mean ± S.E.M. and statistical significance indicated as *P < 0.05.

IDO, like iNOS in murine MSCs, is critical for the immunosuppressive capacity of human MSCs^32^. To study the function of UCHL1 in human MSCs, we firstly detected the UCHL1 expression, which was also found dramatically increased after IFN-γ plus TNF-α stimulation (Fig. 6a). Human MSCs were pretreated with LDN57444, and then co-cultured with activated human peripheral blood mononuclear cells (PBMCs). LDN57444 pretreatment enhanced the immunosuppressive effects of human MSCs on T cell proliferation (Fig. 6b). IFN-γ plus TNF-α-induced IDO expression was also significantly upregulated in human MSCs after LDN57444 pretreatment (Fig. 6c), and the enhanced suppressive effects of LDN57444-pretreated human MSCs on T cell proliferation were reversed by the IDO inhibitor 1-methyl-tryptophan (1-MT) (Fig. 6d). In addition, inhibition of UCHL1 in human MSCs enhanced IFN-γ plus TNF-α-induced phosphorylation of NF-κB p65 and STAT1 at Tyr701 (Fig. 6e), and NF-κB or STAT1 inhibition abolished LDN57444-increased IDO expression (Fig. 6f). Functionally, pretreatment with PDTC or Fludarabine abolished the enhanced suppressive effects on T cell proliferation in LDN57444-pretreated human MSCs (Fig. 6g, h). These data together suggest that UCHL1 also negatively regulates the NF-κB and STAT1 pathway to inhibit the immunosuppressive capacity and IDO expression of human MSCs, indicating an essential role of UCHL1 to regulate the immunosuppressive capacity of both human and murine MSCs.

### UCHL1 promotes proinflammatory cytokines-induced apoptosis of MSCs via downregulation of Bcl-2

**Fig. 7:**
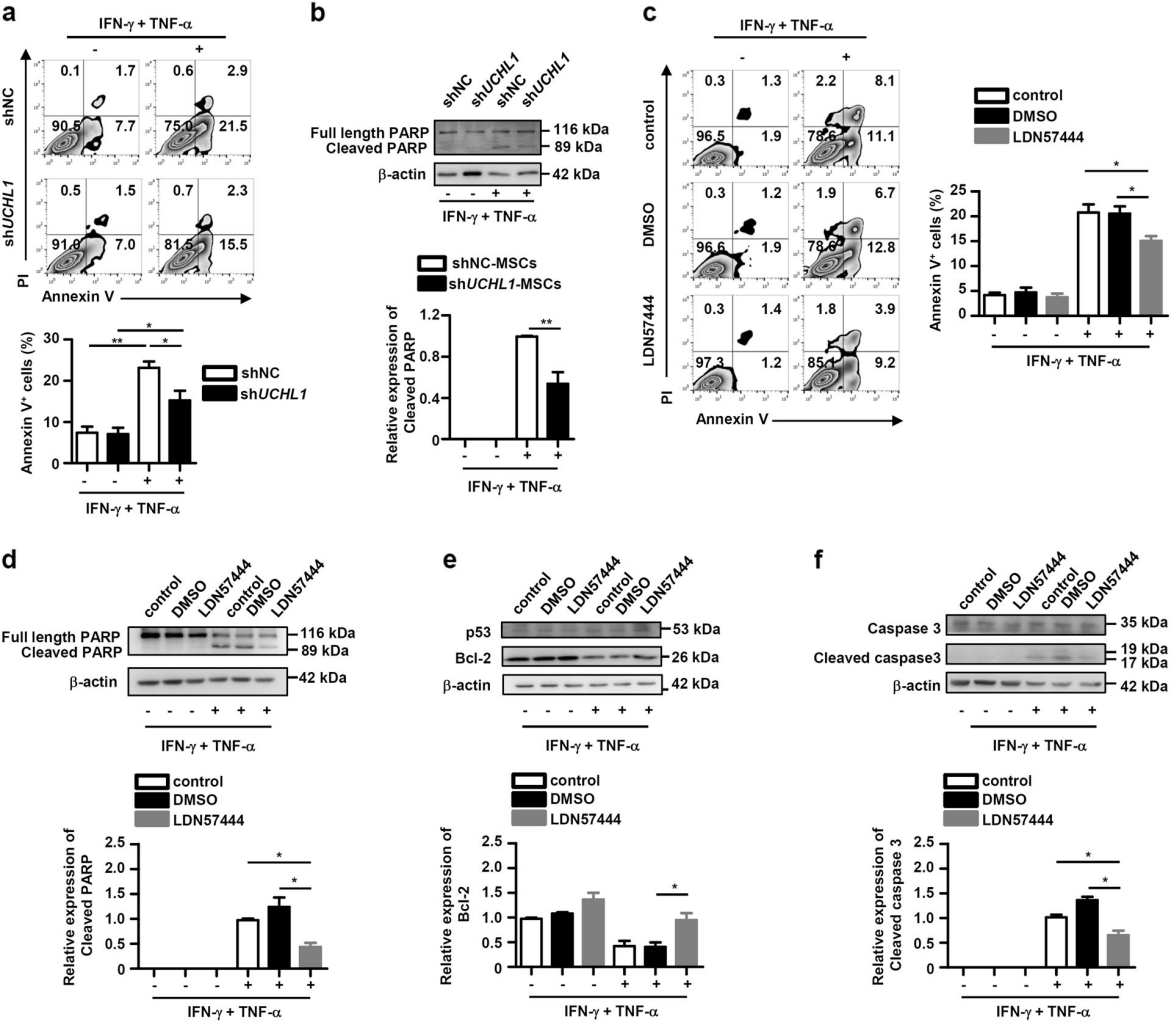
UCHL1 inhibition suppressed IFN-γ plus TNF-α-induced apoptosis of MSCs via upregulation of Bcl-2. shNC-MSCs and sh*UCHL1*-MSCs were treated with or without IFN-γ (50 ng/ml) plus TNF-α (20 ng/ml) for 24 h. **a** Cells were harvested and stained with annexin V/PI, and the percentage of annexin V^+^ MSCs was shown. **b** Cells were harvested and the expression of PARP and cleaved PARP were measured by immunoblotting analysis. Murine MSCs were pretreated with DMSO or LDN57444 (10 μM) for 12 h, and then treated with or without IFN-γ (50 ng/ml) plus TNF-α (20 ng/ml) for 24 h. **c** Cells were harvested and stained with annexin V/PI, and the percentage of annexin V^+^ MSCs was shown. **d** Cells were harvested and the expression of PARP and cleaved PARP were measured by immunoblotting analysis. **e**, **f** The expression of p53, Bcl-2, caspase 3, and cleaved caspase 3 were measured by immunoblotting analysis. Values are shown as mean ± S.E.M. and statistical significance indicated as *P < 0.05 and **P *<* 0.01.

The proinflammatory cytokines, mainly IFN-γ plus TNF-α, not only induce the immunosuppressive capacity, but also are capable of inducing MSC apoptosis^12,13^. To this end, we investigated whether UCHL1 regulated proinflammatory cytokines-induced MSC apoptosis. As shown in Fig. 7a, b, UCHL1 knockdown dramatically inhibited cytokines-induced MSC apoptosis, characterized by significant decrease in the fraction of annexin V^+^ cells and the lower level of cleaved poly ADP-ribose polymerase (PARP) in IFN-γ plus TNF-α-treated sh*UCHL1*-MSCs compared with shNC-MSCs. Consistently, IFN-γ plus TNF-α-induced apoptosis was also suppressed after LDN57444 pretreatment (Fig. 7c, d).

To explore the mechanism of UCHL1-regulated MSC apoptosis, we examined the expression of p53, a known target protein of UCHL1^26^, upon cytokine stimulation. However, there was no obvious difference of p53 protein expression after UCHL1 inhibition (Fig. 7e). Our previous study has demonstrated that the anti-apoptotic protein Bcl-2 modulates caspase activation to inhibit MSC apoptosis under the inflammatory microenvironment^12^. Here, we showed that IFN-γ plus TNF-α stimulation inhibited Bcl-2 expression and promoted the expression of cleaved caspase 3. Interestingly, UCHL1 inhibition markedly inhibited IFN-γ plus TNF-α-induced decrease of Bcl-2 and activation of caspase 3 (Fig. 7e, f), indicating the suppression of IFN-γ plus TNF-α-induced MSC apoptosis. These data suggest that UCHL1 inhibition suppresses MSC apoptosis induced by proinflammatory cytokines via upregulation of Bcl-2.

### Inhibition of UCHL1 in MSCs improves their therapeutic effects on ConA-induced liver injury

**Fig. 8:**
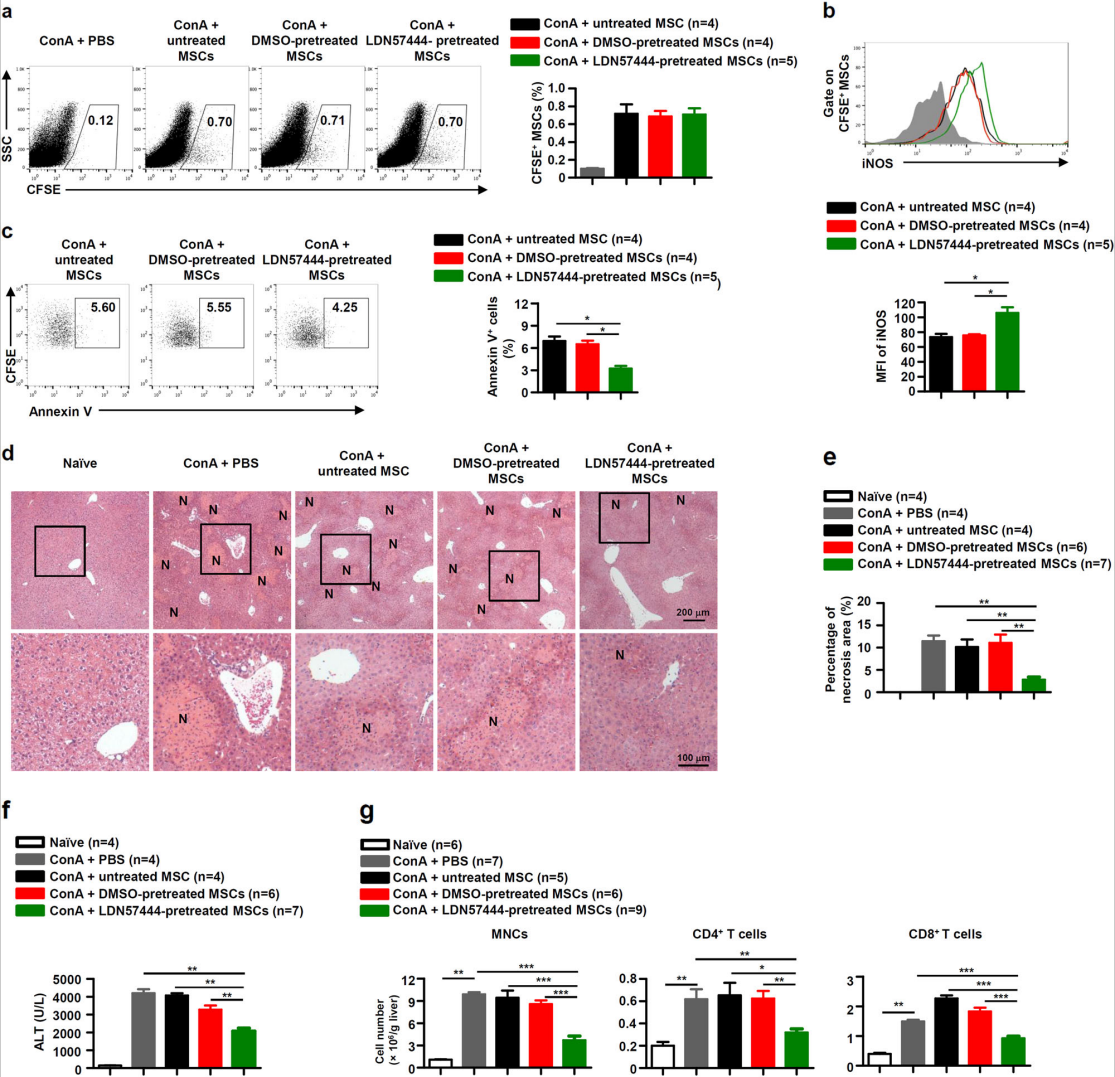
Murine MSCs pretreated with LDN57444 alleviated ConA-induced liver injury. Mice were intravenously injected with ConA (20 mg/kg), untreated, DMSO-pretreated and LDN57444-pretreated MSCs labeled with CFSE or not were transfused immediately. 8 h later, livers and serum were sampled. **a** Percentages of CFSE-labeled MSCs (CFSE^+^ MSCs) in the livers were calculated. **b** The iNOS expression of CFSE^+^ MSCs was determined by flow cytometry and MFI of iNOS in CFSE^+^ MSCs were shown. **c** Cells were harvested and stained with annexin V, and the percentages of annexin V^+^ cells of CFSE^+^ MSCs were shown. **d** Hematoxylin and eosin staining of liver sections. N: necrosis area. **e** Percentages of necrosis area in the livers were calculated. **f** Serum levels of ALT were measured. **g** Absolute numbers of MNCs, CD4^+^ and CD8^+^ T cells in liver tissues were determined by flow cytometry. Values are shown as mean ± S.E.M. and statistical significance indicated as *P < 0.05, **P *<* 0.01, and ***P *<* 0.001.

As UCHL1 inhibition promoted the immunosuppressive capacity and survival of MSCs *in vitro*, we employed the ConA-induced inflammatory liver injury mouse model to explore the effect of UCHL1 inhibition on MSCs. Murine MSCs pretreated with DMSO or LDN57444 were intravenously injected into the mice immediately after ConA administration. Similar to others’ studies, MSCs migrated into the livers of ConA-treated mice (Fig. 8a), and liver injury was scarcely ameliorated in mice administrated with untreated and DMSO-pretreated MSCs^33^. LDN57444 pretreatment had no impact on the migration of MSCs into the injury liver (Fig. 8a). However, the iNOS expression of the injected MSCs in the livers was increased after LDN57444 pretreatment (Fig. 8b), while the apoptosis was inhibited, characterized by the decrease in the fraction of annexin V^+^ cells (Fig. 8c). Expectedly, administration of LDN57444-pretreated MSCs significantly improved the therapeutic effects on liver injury compared with untreated and DMSO-pretreated MSCs. Less liver centrilobular necrosis (Fig. 8d, e) and lower serum alanine aminotransferase (ALT) levels (Fig. 8f) were detected in mice administrated with LDN57444-pretreated MSCs. In addition, we found that there were less mononuclear cells (MNCs), CD4^+^ and CD8^+^ T cells infiltrated in livers of mice administered with LDN57444-pretreated MSCs (Fig. 8g). These results suggest that LDN57444-pretreated MSCs possess the improved capability to alleviate ConA-induced inflammatory liver injury.

## Discussion

MSCs have potent immunosuppressive capacity to inhibit the functions of activated immune cells (e.g., T cells, B cells, dendritic cells, and macrophages), thereby treating various inflammatory diseases^4^. However, the mechanisms regulating the immunosuppressive capacity of MSCs are still poorly understood. We previously elucidated that autophagy occurred in MSCs under the inflammatory microenvironment, and inhibition of autophagy could improve the immunosuppression and survival of MSCs to enhance their therapeutic effects on inflammatory diseases^11,12^. Our previous studies suggested a dual regulation of inflammation on MSC immunosuppression and survival, which affected their therapeutic efficacy^11,12^. In this study, we found that IFN-γ plus TNF-α upregulated the expression of the DUB enzyme UCHL1 in MSCs, while UCHL1 negatively regulated the immunosuppressive capacity and survival of MSCs, thereby affecting their therapeutic effects on ConA-induced inflammatory liver injury.

Recent studies have found that UCHL1 plays important roles in immune responses. For example, human papillomavirus induced UCHL1 expression in keratinocytes, which inhibited the secretion of type I interferon, interleukin-8, and macrophage inflammatory protein-3 to promote the immune escape of human papillomavirus^28^. The DUB enzymes, such as ubiquitin specific peptidase 1, reportedly regulated osteogenic differentiation of MSCs^17^. However, whether the DUB enzymes play roles in MSC immunosuppression is still unknown. In this study, we identified that UCHL1 expression was upregulated in MSCs by stimulation of the proinflammatory cytokines IFN-γ plus TNF-α. Intervening UCHL1 by shRNA knockdown or its inhibitor LDN57444 or overexpression, we firstly found that UCHL1 negatively regulated the immunosuppressive capacity of murine MSCs, characterized by increased iNOS in murine MSCs. The phenomenon that overexpression of UCHL1 could inhibit TNF-α-induced iNOS in vascular endothelial cells, has also been presented by Yoichi *et al.*^31^. Meanwhile, iNOS and its production NO have been demonstrated critical for MSCs to suppress T cell proliferation^33,34^. We found that inhibition of UCHL1 further increased cytokines-induced NO production in murine MSCs, and inhibition of iNOS by L-NMMA markedly abolished the enhanced immunosuppressive capacity of LDN57444-pretreated MSCs. Thus, iNOS plays an important role in UCHL1-mediated modulation of murine MSC immunosuppression. Murine MSCs produce high levels of iNOS, while human MSCs express abundant IDO in response to proinflammatory cytokine stimulation^32^, although both of them can modulate immune responses dependent on effector molecules such as PGE2 and IL-6^8^. We also found that, similar to murine MSC, UCHL1 inhibition enhanced the immunosuppressive capacity by upregulating IDO expression in human MSCs.

Our results also revealed the molecular mechanisms underlying UCHL1-mediated regulation of iNOS expression and immunosuppression of MSCs. NF-κB signaling is critical for IFN-γ-induced the iNOS expression in macrophages and MSCs^30^. The activation of NF-κB is involved in the induction of UCHL1 expression^35^, while UCHL1 can in turn inhibit the activation of NF-κB to suppress the expression of target genes^28,31^. In this study, we found that inhibition of UCHL1 led to decreased expression of IκBα and increased phosphorylation and nuclear translocation of NF-κB p65, resulting in enhanced activation of NF-κB signaling to upregulate iNOS expression in murine MSCs. What is more, we found that activation of STAT1 signaling, another pathway essential for iNOS expression, was also required for the enhanced immunosuppressive capacity in LDN57444-pretreated MSCs. The regulation of UCHL1 on activation of NF-κB and STAT1 signaling also occurred in human MSCs. However, further work will be required to elucidate the exact molecular mechanisms through which UCHL1 affects the activation of STAT1 signaling in MSCs.

Apoptosis is always accompanied with proinflammatory cytokines-induced immunosuppression of MSCs, which impairs their therapy for diseases and is still a challenge for their application^11–13^. Studies showed that UCHL1 could induce apoptosis by stabilizing p53 in breast and hepatocellular carcinoma^26,36^, and promote ovarian cancer cell apoptosis associated with Bcl-2 family proteins-regulated caspase activation^37^. Here, we found that inhibition of UCHL1 suppressed IFN-γ plus TNF-α-induced MSC apoptosis, and this effect was not dependent on p53, the known target protein of UCHL1. UCHL1 inhibition markedly increased Bcl-2 expression and suppressed the expression of cleaved caspase 3. The results suggest that the proinflammatory cytokines upregulate UCHL1, which in turn promotes MSC apoptosis via Bcl-2-regulated activation of caspase 3.

We have found that inhibition of UCHL1 can promote the immunosuppression and survival of MSCs in vitro. We therefore employed a ConA-induced inflammatory liver injury mouse model, a well-established model of liver damage induced by acute immune responses, in which T lymphocytes are identified as the major effector cells^38^. Several studies have reported that human tonsil and murine adipose tissue-derived MSCs can ameliorate the development of ConA-induced liver injury^39,40^. Han et al. firstly found that murine bone morrow MSCs had no effect on this acute inflammatory disease model, while interleukin-17-pretreated MSCs exhibited enhanced therapeutic effects^33^. In the present study, untreated and DMSO-pretreated MSCs had poor therapeutic effects on ConA-induced liver injury, while LDN57444-pretreated MSCs notably alleviated this inflammatory disease.

In summary, our findings demonstrate a critical role of UCHL1 in the dual regulation of inflammatory environment on the immunosuppressive capacity of MSCs: while proinflammatory cytokines empower MSCs to suppress immune responses, these cytokines increase UCHL1 to negatively regulate the immunosuppressive capacity and survival of MSCs. Therefore, modulation of UCHL1 in MSCs may provide a novel strategy to improve MSC-based immunotherapy. Given its potent effects on reducing T cell responses, it will be valuable to investigate whether inhibition of UCHL1 in MSCs may augment their therapeutic efficacy on other T cell-mediated inflammatory disorders.

## Materials and Methods

### Reagents and mice

Recombinant murine and human IFN-γ and TNF-α were purchased from PeproTech (Rocky Hill, USA). Antibodies against β-actin, GAPDH, UCHL1, iNOS, NF-κB p65, Phospho-NF-κB p65, IκBα, histone H3, STAT1, Phospho-STAT1 (Tyr701), IDO, PARP, cleaved PARP, p53, Bcl-2, caspase 3, and cleaved caspase 3 were purchased from Cell Signaling Technology (Danvers, Massachusetts, USA). LDN57444, L-NMMA, Fludarabine, PDTC were purchased from Selleckchem (Houston, USA). ConA, Griess reagents and 1-MT were from Sigma (St Louis, MO, USA). Annexin V/prodium iodide (PI) staining kit were purchased from Life Technologies GmbH (Darmstadt, Germany). C57BL/6 mice were purchased from the Shanghai Laboratory Animal Center of the Chinese Academy of Sciences and maintained under specific pathogen-free conditions in the vivarium of Shanghai Jiao Tong University School of Medicine. All animal procedures were approved by the Animal Welfare and Ethics Committee of Shanghai Jiao Tong University School of Medicine.

### Cells

Murine bone marrow MSCs were isolated as previously described^11,41^. Briefly, murine MSCs were generated from the bone cavity of femurs and tibias of 3-4-week-old C57BL/6 mice. The cells were cultured in DMEM-low glucose containing 10% FBS, 2 mM L-glutamine and 1% Penicillin-Streptomycin (All from Life Technologies GmbH). A single-cell suspension of bone marrow cells was seeded in 100 mm culture dish, non-adherent cells were removed after 24 h, and medium was replenished every 2 days. Cells were used at the 9-16th passages. Human bone marrow MSCs were purchased from Cyagen (Cat. HUXMA-01001, Santa Clara, USA), and used at the 4-8th passages. ‘Stemness’ of murine and human MSCs were determined by their expression of specific cell surface markers, and by their capability to differentiate into adipocytes, osteoblasts, and chondrocytes. MSCs used in each experiment were paired and at the same passage. HEK 293 T were purchased from ATCC (Manassas, USA).

### Real-time PCR

Total RNA was extracted with TRIzol (Life Technologies GmbH) and reverse-transcribed into cDNA with the reverse transcription kit from TaKaRa (Tokyo, Japan). The levels of mRNAs were measured by real-time PCR with SYBR Green reagent from Roche (Natley, New Jersey, USA) and normalized to the mRNA level of β-actin. Primer sequences were as follows: mouse β-actin, sense 5′- CCACGAGCGGTTCCGATG-3′ and antisense 5′-GCCACAGGA TTCCA TACCCA-3′; mouse UCHL1, sense 5′-AGGGACAGGAAGTTAGCCCTA-3′ and antisense 5′-AGCTTCTCCGTTTCAGACAGA-3′; mouse iNOS, sense 5′-TGGAGCGAGTTGTGGATTGT-3′ and antisense 5′-GGGTCGTAATGTCCAGGAAGTA-3′; mouse CXCL9, sense 5′-TGCTACACTGAAGAACGGAGATC-3′ and antisense 5′-CTTCCTTGAACGACGACGACT-3′; mouse CXCL10, sense 5’-GTAAGCTATGTGGAGGTGCG-3′ and antisense 5′-GGAAGATGGTGGTTAAGTTCG-3′; mouse PTGS2, sense 5′-GAAGTCTTTGGTCTGGTGCCT-3′ and antisense 5′-TGCTCCTGCTTGAGTATGTCG-3′; mouse PGE2 sense 5′- TAGTGCCCTCAAGACCTACCTG-3′ and antisense 5′-CTTCCCGCCATACATCTGC-3′; mouse FasL sense 5′-AAGAAGGACCACAACACAAATCTG-3′ and antisense 5′-CCCTGTTAAATGGGCCACACT-3′; mouse CD155 sense 5′-GGGTGGGGATATACGTGTGC-3′ and antisense 5′-GTTCCTCAGATCCTGTTGGGC-3′; mouse CD122 sense 5′-CTCTGTGGATCGAATGGTCA-3′ and antisense 5′-GGCAGCGATAATACCTCCAA-3′. All primers were synthesized by Sangon Biotech (Shanghai, China).

### Immunoblot

Cells were harvested and lysed in the RIPA buffer (Beyotime, Haimen, China) containing PMSF (Beyotime) for 30 min on ice. Lysates were clarified by centrifugation at 15,000×*g* for 30 min. Protein concentration of the supernatant fraction was determined by the Bradford assay (Thermo Fisher Scientific, New Hampshire, USA). Protein samples were diluted in 4× SDS loading buffer (TaKaRa) and heated to 95 °C for 5 min and fractionated in a 10 or 8% SDS-polyacrylamide gel. Proteins were electroblotted onto a polyvinylidene fluoride and incubated for 1 h in 5% bovine serum albumin in Phosphate Buffer Solution (PBS) or nonfat dry milk dissolved in PBS containing 0.1% Tween-20 (PBST) at room temperature. The blotting membranes were incubated with primary antibodies overnight at 4 °C, extensively washed in PBST, incubated with HRP-conjugated secondary antibody (Cell Signaling Technology) for 1 h at room temperature, and washed again with PBST. The blotting membranes were developed with chemiluminescent reagents (Millipore, Billerica, MA, USA) according to the instructions provide by the manufacturer.

### Lentiviral vector construction

Oligonucleotides with the following nucleotide sequences were used for the cloning of shRNA-encoding sequences into a lentiviral vector PLKO.1 puro, a gift from Bob Weinberg (Addgene, Cambridge, USA): mouse UCHL1 (sh*UCHL1*), 5′-CCGGCATGAAGCAGACCATCGGAAACTCGAGTTTCCGATGGTCTGCTTCATGTTTTTG-3′; Scrambled control (shNC), 5′-CCGGCCTAAGGTTAAGTCGCCCTCGCTCGAGCGAGGGCGACTTAACCTTAGGTTTTTG-3′ (Synthetized by Sangon Biotech). High titer lentiviral stocks were produced, and murine MSCs at the 9th passage were infected with scrambled control lentivirus (shNC-MSCs) or lentivirus expressing shRNA inhibiting UCHL1 (sh*UCHL1*-MSCs) according to the manufacturer’s protocol (http://www.addgene.org/tools/protocols/plko/). Cells resistant to puromycin (4 μg/ml) were selected at the 14th passage and used at the 14-16th passages.

### Overexpression

Full-length mouse UCHL1 cDNA were synthesizing in Genscript (Nanjing, China). These cDNAs were subcloned into pcDNA3.1 vectors containing an N-terminal His epitope tag. Murine MSCs were transfected with pcDNA3.1 or pcDNA3.1 containing UCHL1 by using Lipofectamine 2000 (Life Technologies GmbH).

### *In vitro* T cell proliferation assay

Murine MSCs with different treatments were irradiated with 30 Gy from a 137 Cs source as previously described^11^, to inactivate MSCs by inhibiting their proliferation while reserving their immunosuppressive capacity, and then seeded into 96-well plates. Freshly isolated splenocytes (2 × 10^5^ cells/well) from C57/BL6 mice were labeled with 7.5 μM carboxyfluorescein diacetatesuccinimidyl ester (CFSE, Thermo Fisher Scientific) and co-cultured with murine MSCs for 3 days in the presence of mouse anti-CD3/CD28 antibodies (eBiosciences, San Diego, CA, USA), then collected for flow cytometric analysis on a FACS Calibur flow cytometer (BD Biosciences, Franklin Lakes, NJ, USA). Human MSCs with different treatments were irradiated with 30 Gy from a 137 Cs source and seeded into 96-well plates. Freshly isolated PBMCs (2 × 10^5^ cells/well) from healthy volunteers were labeled with 7.5 μM CFSE and co-cultured with human MSCs for 3 days in the presence of human anti-CD3/CD28 antibodies (eBiosciences), then collected for flow cytometric analysis on a FACS Calibur flow cytometer.

### Griess assay

Fifty-microliter culture supernatant fraction of different treated murine MSCs and standards were added to a flat bottomed 96-well plate, and then 50 μL of Griess reagent (Sigma) was added. Read the absorbance at 540 nm after incubation for 15 min in the dark at room temperature, and calculated NO concentrations.

### Induction and detection of murine MSC apoptosis

Murine MSCs were treated with IFN-γ (50 ng/ml) plus TNF-α (20 ng/ml) for 24 h to induced apoptosis as previously described^12.^ For apoptosis analysis, cells were stained with annexin V and prodium iodide according to the manufacturer’s protocol, and then analyzed by flow cytometry on a FACS Calibur flow cytometer.

### ConA-induced liver injury in mice

Male C57BL/6 mice (8-10-week old) were intravenously injected with ConA in PBS at 20 mg/kg to induce inflammatory liver injury. Murine MSCs (1 × 10^6^) were pretreated with DMSO or LDN57444 for 24 h and then intravenously administrated into mice immediately after ConA injection. Mice were killed, and serum and liver tissues were sampled 8 h after ConA injection. 4% paraformaldehyde-fixed liver histological sections were stained with hematoxylin and eosin. Liver mononuclear cells (MNCs) were purified by a percoll gradient and stained with anti-CD4 and anti-CD8a (eBiosciences) for 30 min at 4 °C in staining buffer, and then analyzed by flow cytometry on a FACS Calibur flow cytometer (BD Biosciences). For detection of murine MSCs in the livers of ConA-treated mice, murine MSCs were labeled by preincubation with CFSE and intravenously administrated into mice immediately after ConA injection. MNC suspensions were prepared from liver tissues 8 h after Con A injection and CFSE^+^ MSCs were analyzed by flow cytometry. For iNOS analysis, cells were permeabilized with the Intracellular Fixation and Permeabilization Buffer Set (eBioscience) and stained with anti-iNOS antibody. The secondary antibody was Alexa Fluor 647-conjugated antibody (invitrogen) and the samples were then analyzed by flow cytometry on a FACS Calibur flow cytometer. For apoptosis analysis, cells were stained with annexin V, and then analyzed by flow cytometry on a FACS Calibur flow cytometer.

### Statistical analysis

All measurement data were presented as mean ± S.E.M. Statistical significance was evaluated using unpaired nonparametric test. Significance was expressed as: *P *<* 0.05, **P < 0.01, and ***P *<* 0.001.

## References

[1] Bianco P, Robey PG, Simmons PJ (2008). Mesenchymal stem cells: revisiting history, concepts, and assays. Cell Stem Cell.

[2] Keating A (2012). Mesenchymal stromal cells: new directions. Cell Stem Cell.

[3] Bernardo ME, Fibbe WE (2013). Mesenchymal stromal cells: sensors and switchers of inflammation. Cell Stem Cell.

[4] Uccelli A, Moretta L, Pistoia V (2008). Mesenchymal stem cells in health and disease. Nat. Rev. Immunol..

[5] Shi Y (2010). Mesenchymal stem cells: a new strategy for immunosuppression and tissue repair. Cell Res..

[6] Akiyama K (2012). Mesenchymal-stem-cell-induced immunoregulation involves FAS-ligand-/FAS-mediated T cell apoptosis. Cell Stem Cell.

[7] Krampera M (2013). Immunological characterization of multipotent mesenchymal stromal cells--The International Society for Cellular Therapy (ISCT) working proposal. Cytotherapy.

[8] Wang Y, Chen X, Cao W, Shi Y (2014). Plasticity of mesenchymal stem cells in immunomodulation: pathological and therapeutic implications. Nat. Immunol..

[9] Xu C (2013). miR-155 regulates immune modulatory properties of mesenchymal stem cells by targeting TAK1-binding protein 2. J. Biol. Chem..

[10] Zhang L (2014). SOCS1 regulates the immune modulatory properties of mesenchymal stem cells by inhibiting nitric oxide production. PLoS One.

[11] Dang S (2014). Autophagy regulates the therapeutic potential of mesenchymal stem cells in experimental autoimmune encephalomyelitis. Autophagy.

[12] Dang S (2015). Autophagy promotes apoptosis of mesenchymal stem cells under inflammatory microenvironment. Stem Cell Res. Ther..

[13] Liu Y (2011). Mesenchymal stem cell-based tissue regeneration is governed by recipient T lymphocytes via IFN-gamma and TNF-alpha. Nat. Med..

[14] Reyes-Turcu FE, Ventii KH, Wilkinson KD (2009). Regulation and cellular roles of ubiquitin-specific deubiquitinating enzymes. Annu. Rev. Biochem..

[15] Ma A, Malynn BA (2012). A20: linking a complex regulator of ubiquitylation to immunity and human disease. Nat. Rev. Immunol..

[16] Zinngrebe J, Montinaro A, Peltzer N, Walczak H (2014). Ubiquitin in the immune system. EMBO Rep..

[17] Williams SA (2011). USP1 deubiquitinates ID proteins to preserve a mesenchymal stem cell program in osteosarcoma. Cell.

[18] Zhao L (2011). Tumor necrosis factor inhibits mesenchymal stem cell differentiation into osteoblasts via the ubiquitin E3 ligase Wwp1. Stem Cells.

[19] Chang J (2013). NF-kappaB inhibits osteogenic differentiation of mesenchymal stem cells by promoting beta-catenin degradation. Proc. Natl Acad. Sci. USA.

[20] Dieudonne FX (2013). Promotion of osteoblast differentiation in mesenchymal cells through Cbl-mediated control of STAT5 activity. Stem Cells.

[21] Liu Y, Fallon L, Lashuel HA, Liu Z, Lansbury PT (2002). The UCH-L1 gene encodes two opposing enzymatic activities that affect alpha-synuclein degradation and Parkinson’s disease susceptibility. Cell.

[22] Fang Y, Fu D, Shen XZ (2010). The potential role of ubiquitin c-terminal hydrolases in oncogenesis. Biochim. Biophys. Acta.

[23] Brinkmann K (2013). Ubiquitin C-terminal hydrolase-L1 potentiates cancer chemosensitivity by stabilizing NOXA. Cell Rep..

[24] Li L (2010). The tumor suppressor UCHL1 forms a complex with p53/MDM2/ARF to promote p53 signaling and is frequently silenced in nasopharyngeal carcinoma. Clin. Cancer Res..

[25] Goto Y (2015). UCHL1 provides diagnostic and antimetastatic strategies due to its deubiquitinating effect on HIF-1alpha. Nat. Commun..

[26] Yu J (2008). Epigenetic identification of ubiquitin carboxyl-terminal hydrolase L1 as a functional tumor suppressor and biomarker for hepatocellular carcinoma and other digestive tumors. Hepatology.

[27] Fellenberg J, Lehner B, Witte D (2010). Silencing of the UCHL1 gene in giant cell tumors of bone. Int. J. Cancer.

[28] Karim R (2013). Human papillomavirus (HPV) upregulates the cellular deubiquitinase UCHL1 to suppress the keratinocyte’s innate immune response. PLoS Pathog..

[29] Liu Y (2003). Discovery of inhibitors that elucidate the role of UCH-L1 activity in the H1299 lung cancer cell line. Chem. Biol..

[30] Chen X (2014). The interaction between mesenchymal stem cells and steroids during inflammation. Cell Death Dis..

[31] Takami Y (2007). Ubiquitin carboxyl-terminal hydrolase L1, a novel deubiquitinating enzyme in the vasculature, attenuates NF-kappaB activation. Arterioscler. Thromb. Vasc. Biol..

[32] Ren G (2009). Species variation in the mechanisms of mesenchymal stem cell-mediated immunosuppression. Stem Cells.

[33] Han X (2014). Interleukin-17 enhances immunosuppression by mesenchymal stem cells. Cell Death Differ..

[34] Ren G (2008). Mesenchymal stem cell-mediated immunosuppression occurs via concerted action of chemokines and nitric oxide. Cell Stem Cell.

[35] Guglielmotto M (2012). Abeta1-42-mediated down-regulation of Uch-L1 is dependent on NF-kappaB activation and impaired BACE1 lysosomal degradation. Aging Cell.

[36] Xiang T (2012). The ubiquitin peptidase UCHL1 induces G0/G1 cell cycle arrest and apoptosis through stabilizing p53 and is frequently silenced in breast cancer. PLoS One.

[37] Jin C (2013). UCHL1 is a putative tumor suppressor in ovarian cancer cells and contributes to cisplatin resistance. J. Cancer.

[38] Wang HX (2012). Immune mechanisms of concanavalin A model of autoimmune hepatitis. World J. Gastroenterol..

[39] Higashimoto M (2013). Adipose tissue derived stromal stem cell therapy in murine ConA-derived hepatitis is dependent on myeloid-lineage and CD4+T-cell suppression. Eur. J. Immunol..

[40] Ryu KH (2014). Tonsil-derived mesenchymal stem cells alleviate concanavalin A-induced acute liver injury. Exp. Cell Res..

[41] Zhang Y (2014). Mesenchymal stem cells alleviate bacteria-induced liver injury in mice by inducing regulatory dendritic cells. Hepatology.

